# *GSTM1* and *GSTT1 double null* genotypes determining cell fate and proliferation as potential risk factors of relapse in children with hematological malignancies after hematopoietic stem cell transplantation

**DOI:** 10.1007/s00432-021-03769-2

**Published:** 2021-09-09

**Authors:** Simona Jurkovic Mlakar, Satyanarayana Chakradhara Rao Uppugunduri, Tiago Nava, Vid Mlakar, Hadrien Golay, Shannon Robin, Nicolas Waespe, Mohamed Aziz Rezgui, Yves Chalandon, Jaap Jan Boelens, Robert G. M. Bredius, Jean-Hugues Dalle, Christina Peters, Selim Corbacioglu, Henrique Bittencourt, Maja Krajinovic, Marc Ansari, the paediatric diseases working party of the European society for blood and marrow transplantation

**Affiliations:** 1 CANSEARCH Research Platform in Paediatric Oncology and Haematology, Department of Paediatrics, Gynecology and Obstetrics, Faculty of Medicine, University of Geneva, Geneva, Switzerland; 2 Division of Paediatric Oncology and Haematology, Department of Women, Children and Adolescents, Pediatric Oncology and Hematology Unit, Geneva University Hospitals and University of Geneva, Rue Willy-Donzé 6; Bureau 5-507, 1211 Genève, Switzerland; 3 Institute of Social and Preventive Medicine, University of Bern, Bern, Switzerland; 4 Charles-Bruneau Cancer Center, CHU Sainte-Justine Research Center, Montreal, QC Canada; 5 Division of Haematology, Department of Oncology, Geneva University Hospital and Faculty of Medicine, University of Geneva, Geneva, Switzerland; 6 Paediatric Blood and Marrow Transplantation Program, University Medical Center Utrecht, Utrecht, The Netherlands; 7 Department of Pediatrics, Leiden University Medical Center, Leiden, The Netherlands; 8 Paediatric Haematology Department, Robert Debré Hospital, Assistance Publique, Hôpitaux de Paris, Paris, France; 9 St Anna Children’s Hospital, Department of Pediatrics, Medical University Vienna, Vienna, Austria; 10 Department of Pediatric Hematology, Oncology and Stem Cell Transplantation, University of Regensburg, Regensburg, Germany; 11 Department of Paediatrics, Faculty of Medicine, University of Montreal, Montreal, QC Canada; 12 Present Address: Clinical Pharmacology Unit, CHU Sainte-Justine, Montreal, QC Canada; 13 Department of Pharmacology, Faculty of Medicine, University of Montreal, Montreal, QC Canada

**Keywords:** *Null* genotypes of glutathione S-transferases, Acute leukemia, Hematological malignancies, Hematopoietic stem cell transplantation, Post-transplant relapse, Busulfan resistance

## Abstract

**Purpose:**

This study aimed to retrospectively evaluate the genetic association of *null* variants of glutathione S-transferases *GSTM1* and *GSTT1* with relapse incidence in children with hematological malignancies (HMs) undergoing busulfan (BU)- containing allogeneic hematopoietic stem cell transplantation (HSCT) and to assess the impact of these variants on BU-induced cytotoxicity on the immortalized lymphoblastoid cell lines (LCLs) and tumor THP1 *GST* gene-edited cell models.

**Methods:**

*GSTM1- and GSTT1-null* alleles were genotyped using germline DNA from whole blood prior to a conditioning BU-based regimen. Association of *GSTM1-* and *GSTT1-null* variants with relapse incidence was analyzed using multivariable competing risk analysis. BU-induced cell death studies were conducted in *GSTs*- *null* and *non-null* LCLs and CRISPR–Cas9 gene-edited THP1 leukemia cell lines.

**Results:**

Carrying *GSTM1/GSTT1 double null* genotype was found to be an independent risk factor for post-HSCT relapse in 86 children (adjusted HR: 6.52 [95% Cl, 2.76–15.42; *p* = 1.9 × 10^–5^]). BU-induced cell death preferentially in THP1^*GSTM1(non−null)*^ and LCLs^*GSTM1(non−null)*^ as shown by decreased viability, increased necrosis and levels of the oxidized form of glutathione compared to *null* cells, while *GSTT1 non-null* cells showed increased baseline proliferation.

**Conclusion:**

The clinical association suggests that *GSTM1*/*GSTT1 double null* genotype could serve as genetic stratification biomarker for the high risk of post-HSCT relapse. Functional studies have indicated that *GSTM1* status modulates BU-induced cell death. On the other hand, GSTT1 is proposed to be involved in baseline cell proliferation.

**Supplementary Information:**

The online version contains supplementary material available at 10.1007/s00432-021-03769-2.

## Background

Survival rates of children with hematological malignancies (HMs) undergoing allogeneic hematopoietic stem cell transplantation (HSCT) have improved over the years achieving 91% estimated 2-year overall survival. The improvement is mainly attributed to reduced HSCT-related toxicity and mortality. The incidence of post-HSCT relapse remains a significant complication and varies from 12 to 33% after 2 years (Peters et al. 2021). Risk factors that influence transplant success are on the one hand host- and disease related, such as disease genetics and remission status before HSCT, and on the other hand, transplant related, such as conditioning regimen and treatment-related toxicities including for example severe graft-versus-host disease (GvHD), sinusoidal obstruction syndrome (SOS), and infections (Barrett and Battiwalla 2010; Hamilton and Copelan 2012; Shah et al. 2014).

A bifunctional alkylating agent busulfan (BU) is still often used in conditioning regimens prior to HSCT in children and adolescents (Philippe et al. 2016) and is commonly administered along with other chemotherapeutics, e.g., cyclophosphamide (CY) and fludarabine (FLU) (Ciurea and Andersson 2009; Hao et al. 2020). At least in acute myeloid leukemia (AML), BU has shown lower long-term adverse effects, consequently replacing total body irradiation (TBI) in the conditioning regimen (Lee et al. 2020). In acute lymphoblastic leukemia (ALL), although the recently published results showed lower rates of relapse after TBI-containing conditioning, the results obtained with BU in association with FLU and thiotepa were encouraging and indicate an opportunity to find genetic subgroups of patients who might benefit from the TBI-free conditioning (Peters et al. 2021).

BU is metabolized via conjugation with glutathione (GSH) in the liver, which is predominantly catalyzed by glutathione S-transferase alpha1 (GSTA1) (Czerwinski et al. 1996). In hematopoietic cells (HCs), where GSTA1 is not expressed (Czerwinski et al. 1997), other GST isoenzymes, particularly Mu1 (GSTM1, 46% of the BU conjugating activity of GSTA1 (Czerwinski et al. 1996)) might play the most important role. The role of GSTT1 in BU conjugation is not yet known, but has been mostly reported to have combined effects with GSTM1 on clinical outcomes (Kim et al. 2011; Myers et al. 2017). In addition to their protective role of the conjugation of BU in HCs, they might contribute to multiple cellular processes such as regulation of cell proliferation and apoptosis through the interaction with protein kinases such as apoptosis signal-regulating kinase 1 (ASK1). Under stress conditions, the interaction of the GSTM1:ASK1 complex is dissociated and results in activation of ASK1 that activates the c-Jun N-terminal kinase (JNK) and mitogen-activated protein kinase p38 (MAPK p38) pathways, leading to upstream cytokine- and stress-induced apoptosis (Board and Menon 2013; Tew and Townsend 2012). However, the impact of apoptosis through kinases on BU-dependent cytotoxicity is poorly understood and even less whether those *GST* genes naturally knocked down might interfere in the post-HSCT relapse potential.

*GSTM1* and *GSTT1* genes can be homozygously deleted (presented as *GSTM1-null* and *GSTT1-null*) and thus completely deprived of the enzyme activities in a high percentage of individuals (the average % in Europe are 51 and 19, respectively) (Saitou and Ishida 2015). In AML adult patients, Weiss et al. (Weiss et al. 2007) showed a perfect concordance of those variants in malignant and germline DNA, which suggests that the germline genotype drives protein expression in malignant cells. Although these variants have been associated with a higher risk of leukemia development (Li et al. 2018), there are conflicting reports on the association of the *GSTM1-null* and *GSTT1-null* variants with relapse in patients with HMs (Balta et al. 2003; Franca et al. 2012; Stanulla et al. 2000; Takanashi et al. 2003). To date, there is no evidence available for the association of germline *GSTM1-null* and *GSTT1-null* variants with post-HSCT relapse in children with HMs.

Because GSTM1 and GSTT1 are the main remaining GSTs in HCs, we hypothesized that the absence of either or both proteins should affect BU cytotoxicity through conjugation-dependent or -independent ways, interfering in the HSCT outcomes. Hence, a genetic association study based on germline *GSTT1-* and *GSTM1-null* variants was undertaken. Further, we conducted in vitro functional analyses to understand the role of these variants in survival and BU-induced apoptosis and necrosis of the immortalized and tumor lymphoblastoid cell lines (LCLs).

## Materials and methods

### Clinical association study

#### Patients and treatment

Pediatric patients with ALL, AML or myelodysplastic syndrome (MDS) who had undergone allogeneic HSCT between 2000 and 2013 were enrolled in the study. The Institutional Review Board or ethics committees approved the study and all patients and/or parents provided informed consent. The present study is a subset of the multicentric study under the umbrella of the European Society for Blood and Marrow Transplantation (EBMT) (Clinicaltrials.gov identifier: NCT01257854) (Ansari et al. 2017).

I.v. BU (Busulfex, Otsuka Pharmaceuticals, Saint-Laurent, Montreal, QC, Canada or Busilvex, Pierre Fabre Laboratory, Paris, France) administration was given as a 2 h infusion to the patients, every 6 h for a total of 16 doses. The first BU dose was age and weight based and pharmacokinetic (PK)-guided dose adjustment was performed to obtain a cumulative area under the curve (CumAUC) between 59.2 and 98.56 mg*h/L as reported previously (Ansari et al. 2017)*.*

The primary diagnosis of HMs was made at the referring institution. Patients were considered to be in remission after chemotherapy if they presented < 5% blasts in the normal cellular bone marrow. Relapse in MDS was defined as > 5% and ≤ 20% of blasts at the bone marrow examination after engraftment and/or reappearance of major dysplastic features associated with cytopenias and/or mixed chimerism > 5% and/or detection of the same cytogenetic abnormality present at diagnosis. Relapse in AML and ALL was defined as the presence of blasts in the bone marrow > 5%, confirmed by flow cytometry; detection of the gene fusion present at diagnosis; or according to minimal residual disease (MRD) results after the transplantation if available. Disease remission status was defined by the number of bone marrow remission or relapse events before HSCT.

Cumulative relapse incidence, event-free survival (EFS), and overall survival (OS) were defined according to the standard guidelines of EBMT and as detailed in our recent report (Ansari et al. 2017). EFS was calculated from the time of transplant until death, relapse, or graft failure, whichever occurred first. OS was calculated from the time of transplant until death from any cause.

#### Genotyping and statistical analysis

Genotyping of *GSTM1-null* and *GSTT1-null* variants was performed on germline DNA, extracted from whole blood or peripheral mononuclear cells of all patients before the first HSCT as described by Lin et al. (1998).

Pearson Chi-square test was used to analyze the differences in demographics between groups with and without *GST-null* variants. Estimated cumulative relapse incidence by competing risk analysis with non-relapse mortality as a competing event and the difference among groups were estimated by Gray’s test (Gray 1988). The Fine–Gray model was used for competing risk regression in multivariable analysis to obtain adjusted *p* values for all the variables in relation to the genotype groups (Fine and Gray 1999). The potential risk factors with a *p* value ≤ 0.25 in the univariable competing risk analysis were retained in the multivariable analysis by including the *GST* genotype factor with the lowest *p* value. The final multivariable analysis included: diagnosis (ALL, AML and MDS), disease status [1st complete remission (CR), a higher degree of CRs and absence of CR], conditioning regimen (standard regimen with two alkylating agents and intensified regimen with three alkylating agents), AUC after the first dose of busulfan (1st BU dose AUC categorized into below 3.7, between 3.7 and 6.16, and above 6.16 mg*h/L) and BU CumAUC (below 59.2, between 59.2 and 98.6, and above 98.6 mg*h/L) as categorical variables. Cumulative incidences of OS and EFS were estimated in relation to the genotype groups, using Kaplan–Meier framework and log-rank test. Univariate Cox regression was used to estimate hazard ratios.

All statistical analyses on clinical data were performed using SPSS (RRID: SCR_002865, Version 24.0. Armonk, NY: IBM Corp.) and R Project for Statistical Computing (version 3.6.2, RRID: SCR_001905) with Rcmdr package (version 2.6.1). Statistical power calculations according to GST variants were conducted in G*Power–Statistical Power Analyses for Windows and Mac, version 3.1.9.2 (RRID: SCR_013726; Dusseldorf, Germany).

### In vitro functional studies of the associated variants

#### Cell models design and cell characterization

A set of 56 immortalized non-malignant lymphoblastoid cell lines (LCLs), acquired in 2012 from International HapMap Consortium’s CEPH Families Reference Panel 142,011/147712 (Coriell Cell Repository, Camden, NJ, USA), and a human monocytic leukemia cell line (THP1; acquired in 2018 from ATCC, Cat# TIB-202, RRID: CVCL_0006; Manassas, Virginia, USA), derived from a 1-year-old patient, were used for baseline and BU-induced functional assessment of *GSTM1-null* and *GSTT1-null* variants. The cells were immediately stored at − 196 °C and were not used prior to the start of experiments. The cell lines were thawed and cultured in Roswell Park Memorial Institute Medium (RPMI) 1640 medium (Gibco, Carlsbad, CA) supplemented with 10% fetal bovine serum (HyClone, South Logan, UT) and 1% penicillin–streptomycin (Gibco) and incubated at 37 °C, 5% CO_2_-humidified atmosphere according to the manufacturer’s recommendations. The number of passages between thawing and use in each in vitro experiment achieved the range between 5 and 15 times. The IDs of investigated LCLs used for each particular in vitro experiment are listed in Supplementary Table 1.

THP1 *GSTM1*- and *GSTT1*-knockout cell lines (THP1^*GSTM1(−/−)*^ and THP1^*GSTT1(−/−)*^) were prepared from parental THP1 representing *non-null* genotype for *GSTM1* (THP1^*GSTM1(*+*/*+*)*^*)* and *GSTT1* (THP1^*GSTT1(*+*/*+*)*^*)* using CRISPR/Cas9 gene-editing method. Plasmid PX458 containing 5’-*TGATACTGGGGTACTGGGAC*-3’ gRNA (*GSTM1*) or 5’-*TGAAGGACGGGGACTTCACC*-3’ gRNA (*GSTT1*) (prepared by GeneScript, The Netherlands) was transfected into THP1 cells. 10,000 cells were fluorescence-activated cell sorted (FACS) in 24-well plates based on the presence of green fluorescence protein (GFP) 48 h post-transfection. After 48–72 h of recovery, THP1 cells were single-cell cloned in 96-well plates using FACS. Gene-modified clones were genotyped for the presence of deleterious mutations using Sanger sequencing and confirmed by Western blot for the success of gene knockout. Five clones of the same genotype were pooled in a population.

DNA and proteins of the selected cell lines (LCLs, THP1^*GSTM1(−/−)*^ and THP1^*GSTT1(−/−)*^ cell models) were extracted using DNeasy Blood and Tissue Kit (Qiagen, Hilden, Germany) and standard protein extraction protocol for western blot using RIPA lysis buffer (Sigma-Aldrich, Germany), respectively. The intracellular concentration of extracted proteins was measured using Bradford assay from Bio-Rad (Hercules, CA) according to the manufacturer’s recommendations. Aliquots containing 20 μg of proteins, sample reducing agent and LDS sample buffer (Thermo Fisher Scientific, USA) were subjected to electrophoresis by using Invitrogen Novex Tris–Glycine Gels (Thermo Fisher Scientific, USA). Dry transfer to a nitrocellulose membrane was performed with the iBlot dry blotting system (ThermoFisher Scientific, USA). A membrane was blocked using 5% milk in PBS and 0.05% Tween 20. The following primary antibodies were used for protein labeling: ß-Actin Mouse monoclonal antibody (Abcam Cat# ab6276, RRID: AB_2223210); GSTP1 Monoclonal Antibody Rabbit (DSHB Cat# CPTC-GSTP1-1, RRID: AB_2617266); GSTM1 Monoclonal Antibody Mouse (Thermo FisherScientific Cat# MA5-17,085, RRID: AB_2538556) and GSTT1 Polyclonal antibody Rabbit (Thermo FisherScientific Cat# PA5-22,011, RRID: AB_11154445). Lumi-Light WB Substrate (Roche, CH) was used for the detection of the secondary antibody linked with horseradish peroxidase (HRP). Band intensities were identified using Syngene G-Box System (Syngene, Frederick, MD, USA).

The glutathione transferase activity (Glutathione S-transferase [GST] Assay Kit, Sigma-Aldrich, USA) was measured on cell lysates obtained from one million cells (THP1^*GSTM1(−/−)*^ and THP1^*GSTM1(*+*/*+*)*^) by measuring absorbance at 340 nm every minute for 10 min in a 200 μl well of a 96-well plate using Spectramax ID3 Multi-Mode microplate reader (Molecular Devices, USA) according to the manufacturer’s recommendations. GST activity was calculated as the following: [Δ340nm (min) * total volume of the reaction (ml)]/[5.3 mM^−1^ * volume of enzyme] corrected according to the protein concentration in mg/ml obtained by using a Pierce™ BCA Protein Assay Kit (ThermoFisher Scientific, USA) according to the manufacturer’s guidelines.

The results of the characterization of CRISPR–Cas9 THP1 gene-edited cell models with target proteins (GSTM1, GSTT1 and GSTP1) and GST activity are presented in Supplementary Fig. 1 (A–C).

DNA samples from LCLs were genotyped for *GSTM1-null* and *GSTT1-null* variants using multiplex real-time PCR amplification in the presence of SYBR Green I and genotype discrimination by melting curve analysis in a StepOnePlus™ Real-Time PCR System (Applied Biosystems™, Foster City, CA, USA) with *BCL2* (*BCL2 apoptosis regulator*) gene as an internal control as described earlier (Marin et al. 2010). The genotyping method used cannot differentiate the heterozygous individuals from homozygous *non-null* carriers (furtherly marked as *GSTM1(* +*)* and *GSTT1(* +*)*) except when using Sanger sequencing.

#### Cell viability, apoptosis, necrosis, GSSG/GSH measurements, and caspase activities

Intracellular ATP concentrations (CellTiter 2.0 Luminescent Cell Viability Assay (Promega Corporation, Madison, WI)) were screened in 56 LCLs and CRISPR–Cas9 gene-edited cell models: THP1^*GSTM1(−/−)*^, THP1^*GSTM1(*+*/*+*)*^, THP1^*GSTT1(−/−)*^, THP1^*GSTT1(*+*/*+*)*^; at 48 h of treatment with 100, 200, 400, 800 and 1600 µM concentrations of BU (Sigma-Aldrich, Germany) reconstituted with DMSO (Sigma), 1% of DMSO (as control) and at baseline (medium only). For validation of the first screening, ATP-independent cell viability follow-up (72 h) was performed at BU concentrations of 100, 250 and 500 µM in three *GSTM1(* +*)* and four *GSTM1(-/-)* LCLs (Supplementary Table 1) and at baseline in THP1^*GSTT1(−/−)*^ and THP1^*GSTT1(*+*/*+*)*^ using RealTime-Glo^MT^ Cell Viability Assay (Promega, USA).

Annexin V/PI assay (BD Biosciences) was used to measure live, early and late apoptotic and necrotic cells. Prior to FACS, ten *GSTM1(* +*)* and ten *GSTM1(-/-)* LCLs (Supplementary Table 1) were treated for 48 h with BU (1% DMSO) at 250, 500 and 1000 µM and two samples of each cell line were used as controls (1% DMSO and untreated). One million cells were labeled according to the manufacturer’s protocol. FACS analysis was performed using the CyAN ADP system (Beckman Coulter, UK). Results were analyzed by Kaluza analysis software, version 1.3 (Beckman Coulter, UK). Apoptosis and necrosis were followed for 72 h in six *GSTM1(* +*)* and six *GSTM1(-/-)* LCLs (Supplementary Table 1) treated with 500 µM BU using RealTime-Glo ^MT^Annexin V Apoptosis and Necrosis Assay (Promega).

Concentrations of the total (GSH_T_) (GSH-Glo Glutathione Assays, Promega, USA) and oxidized intracellular glutathione (GSSG) (GSH/GSSG-Glo Glutathione Assays, Promega, USA) were measured according to the manufacturers’ recommendations. Prior to measurement, cells from five *GSTM1(* +*)* and five *GSTM1(−/−)* LCLs and CRISPR–Cas9 gene-edited THP1 cell models were incubated for 48 h at 500 µM BU and 1% DMSO (control). Results are expressed as the relative proportion of GSSG to GSH_T_.

Caspase-Glo 3/7 assay (Promega) was used to measure the total activity of caspases-3 and -7 in a subset of 12 LCLs (Supplementary Table 1) and CRISPR–Cas9 gene-edited THP1 cell models at 48 h BU post-treatment (250, 500 and 1000 μM).

Chemiluminescent signals were measured using Victor3 (Perkin Elmer, Inc., USA). All BU-based data were normalized relative to the negative controls with 1% DMSO.

#### Statistical analyses in in vitro functional studies

The cell-based experiments (IC_50_ distribution, end-point apoptosis and necrosis, real-time monitoring of viability, apoptosis and necrosis, Caspase3/7 activities; and [GSSG/GSH_T_] ratios) were performed at least in duplicate and results are reported as observed means ± SD stratified by *GST-null* and *GST-non-null* variants. Statistical differences between genotypes were assessed using Mann–Whitney, *t* tests, or two-way ANOVA according to the normality of the distribution and compared to untreated controls using GraphPad Prism 7 software (RRID: SCR_002798). We considered *p* < 0.05 to be statistically significant in all analyses.

## Results

### *GSTM1- and GSTT1-double null* genotypes are associated with higher relapse incidence

Eighty-six children with malignancies aged 5 months–18 years (female/male, 44/42), who received myeloablative conditioning containing four-times-daily i.v. BU followed by HSCT, were enrolled in this study. The patients’ baseline characteristics at the time of their HSCT are summarized in Table 1. The number of patients who had experienced relapse was 16 (18.6% of included patients) with the median time to onset 203 days (range 35 to 817) and 12 (14.0%) patients died with the median time to onset 221 days (range 15 to 979). The median CumAUC of BU achieved 56.96 mg*h/L (concentration range 30.50–115.23 mg*h/L).

**Table 1:** Demographic and transplantation characteristics of pediatric patients at the time of HSCT and events follow-up

Variables	*N* (%)
Sex	
Male	42 (48,8)
Female	44 (51,2)
Ethnicity	
Caucasian	68 (79,1)
Other	18 (20,9)
Diagnosis	
ALL	12 (14,0)
AML	43 (50,0)
MDS	31 (36,0)
Stem cell source	
BM	35 (40,7)
CB	47 (54,7)
PBSCs	4 (4,7)
Regimen conditioning	
Busulfan/cyclophosphamide	64 (74,4)
Busulfan/melphalane	2 (2,3)
Busulfan/cyclophosphamide/melphalane	13 (15,1)
Busulfan/cyclophosphamide/etoposide	7 (8,1)
Serotherapy	
No	31 (36,0)
ATG	55 (64,0)
HLA match compatibility	
MRD	28 (32,6)
MUD	19 (22,1)
MMRD	3 (3,5)
MMUD	36 (41,9)
Disease phase	
CR1	38 (44,2)
CR2	10 (11,6)
CR3 or more*	9 (10,5)
Never treated	24 (27,5)
ND	5 (5,8)
Intensity of conditioning^#^	
2	66 (76,7)
3 or more	20 (23,3)
	Median (range)
Age at HSCT (years)	6,5 (0,5–18,2)
Weight (kg)	24,5 (6,0–87,9)
Height (cm)	122,5 (51,0–183,0)

*ALL* acute lymphoblastic leukemia; *AML* acute myeloid leukemia; *BM*, bone marrow; *BU*, busulfan; *CB* cord blood; *CR1* first complete remission; *CR2* second complete remission; *CR3* third complete remission; *HLA* identical sibling; *MDS* myelodysplastic syndrome; *MMUD* non-identical unrelated; *MMRD* non-identical related; *MUD* identical unrelated; *MRD* identical related; *ND* no data; *PBSCs* peripheral blood stem cells

*Disease phase “CR3 or more” included all patients either in CR3 or more or in partial remission or those with > 10% of circulating myeloblasts before conditioning

^#^2 alkylating agents (busulfan with cyclophosphamide or melphalan) and 3 agents (busulfan/cyclophosphamide with melphalan or etoposide)

The enrolled patients were from CHU St Justine (Montreal, Quebec, Canada), The Hospital for Sick Children (Toronto, Ontario, Canada), Robert Debre University Hospital (Paris, France), Leiden University Medical Center (Leiden, Netherlands) and Geneva University Hospital (Geneva, Switzerland)

Regarding the genotype frequency, 49 patients were *GSTM1-null* (57.0%), 24 *GSTT1-null* (27.9%) and 9 had *null* genotypes in both *GSTM1* and *GSTT1* genes (10.5%). Characteristics of these patients according to *GSTM1-null* and *GSTT1-null* variants are shown in Supplementary Table 2.

**Fig. 1:**
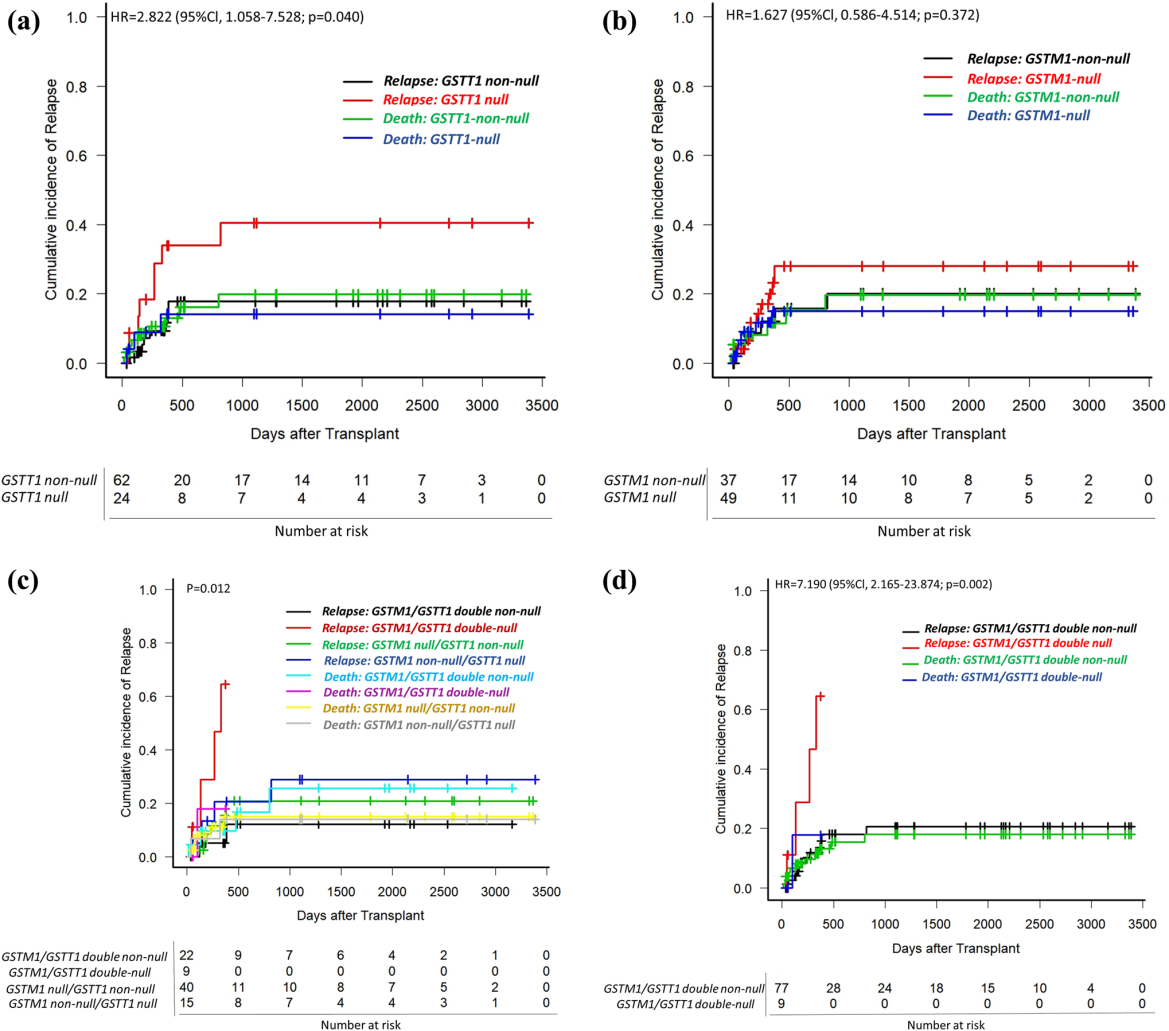
Cumulative incidence plots of relapse in univariable competing risk analyses stratified by *GST-null* variants. Results are plotted for (**A**) *GSTT1(−/−)* group versus groups *GSTT1(* ±*)* and *GSTT1(*+ */* +*)* together presented as *GSTT1(* +*)*; (**B**) *GSTM1(−/−)* group versus groups *GSTM1(* ±*)* and *GSTM1(*+ */* +*)* together presented as *GSTM1(* +*)*; (**C**) combined *GSTM1(−/−)/GSTT1(−/−)* versus other genotype combinations [*GSTM1(−/−)/GSTT1(* +*), GSTM1(* +*)/GSTT1(−/−)* and *GSTM1(* +*)/GSTT1(* +*)*]; (**D**) combined *GSTM1(−/−)/GSTT1(−/−)* versus grouped other genotype combinations [*GSTM1(* +*)/GSTT1(* +*)*]. *p* values for the difference in cumulative incidence of relapse were calculated with Gray’s test with death as a competing event. HR, hazard ratio; *GSTM1-null* and *GSTT1-null* are presented as *GSTM1(−/−)* and *GSTT1(−/−)*, respectively. *GSTM1 non-null* and *GSTT1 non-null* genotypes are presented as *GSTM1(* +*)* and *GSTT1(* +*)*, respectively

Relapse was associated with *GSTT1-null* compared to *GSTT1-non-null* subgroups (42.1% vs 16.1%) in the univariable analysis (*p* = 0.04, Fig. 1A, Table 2A). The *GSTM1-null* was not associated with relapse (Fig. 1B, Table 2A). However, patients carrying *null* genotypes in both *GSTM1* and *GSTT1* genes showed significantly increased risk of relapse compared to other genotype subgroups ([*GSTM1-non-null/GSTT1-non-null, GSTM1-non-null/GSTT1-null and GSTM1-null/GSTT1-non-null*]*; p* = 0.012, Fig. 1C, Table 2A) and this risk remained significant when other genotype subgroups were grouped together (using a gene–gene interaction model; 77.8% vs. 19.0%; *p* = 0.002, Fig. 1D, Table 2B). Significantly lower EFS was observed in the group of patients carrying (−/−) alleles in both *GSTM1* and *GSTT1* genes in comparison to others (54.2% vs. 11.1%, *p* < 0.001, Supplementary Fig. 2A). When gene variants were analyzed independently, none of them affected the EFS (data not presented). OS was not significantly associated with *GST-null* variants (using gene–gene interaction model; 67,1% vs. 37,0%, *p* = 0.401, Supplementary Fig. 2B).

**Table 2:** Relationship between *GSTM1-null* and *GSTT1-null* variants and other risk factors with a cumulative incidence of relapse in univariable and multivariable competing risk analyses (Fine–Gray’s test)

A)			
Covariable	Univariable analysis		
	HR	95% Cl	*p* value (C Risk)
GSTM1 genotype			
			0.372
* GSTM1(* +*) *^*(N*=*37)*^	*Reference*	1	
* GSTM1(−/−) *^*(N*=*49)*^	1.627	(0.586–4.514)	
GSTT1 genotype			
			**0.040**
* GSTT1(* +*)*^*(N*=*62)*^	*Reference*	1	
* GSTT1(−/−)*^*(N*=*24)*^	2.822	(1.058–7.528)	
GSTM1 and GSTT1 genotypes (4 groups)			
			**0.012**
* GSTM1(* +*) and GSTT1(* +*)*^*(N*=*22)*^	*Reference*	1	
* GSTM1(−/−) and GSTT1(−/−)*^*(N*=*9)*^	12.836	(2.218–74.274)	
* GSTM1(−/−) and GSTT1(* +*)*^*(N*=*40)*^	1.879	(0.378–9.354)	
* GSTM1(* +*) and GSTT1(−/−)*^*(N*=*15)*^	2.614	(0.478–14.300)	

B)						
Covariable	Univariable analysis			Multivariable analysis		
	HR	95% Cl	*p* value (CRisk)	HR	95% Cl	*p* value (CRRisk)
*GSTM1 and GSTT1* genotypes (2 groups)						
* GSTM1(* +*)/GSTT1(* +*) and GSTM1(−/−)/GSTT1(* +*) and GSTM1(* +*) /GSTT1(−/−)*^*(N*=*77)*^	Reference	1	**0.002**	Reference	1	**1.9 × 10**^**–5**^
* GSTM1(−/−) and GSTT1(−/−)*^*(N*=*9)*^	7.190	(2.165–23.874)		6.521	(2.758–15.420)	
Diagnosis						
ALL	Reference	1	0.232	Reference	1	0.093
AML	1.077	(0.296–3.925)				
MDS	0.384	(0.077–1.902)		0.503	(0.225–1.121)	
Cum_AUC (mg*h/L)#						
< 59.2	Reference	1	0.062	Reference	1	0.220
59.2–98.6	3.891	(1.327–11.409)		3.947	(0.432–36.040)	
> 98.6	1.741	(0.201–15.055)				
AUC_1stDose (mg*h/L)						
< 3.7	Reference	1	0.090	Reference	1	0.410
3.7–6.16	0.729	(0.089–5.983)		0.391	(0.041–3.737)	
> 6.16	2.230	(0.276–17.991)				
Intensity of conditioning						
2	Reference	1	0.134	Reference	1	0.100
3 or more	0.382	(0.086–1.687)		0.338	(0.091–1.248)	
Disease phase						
CR1	Reference	1	0.166	Reference	1	0.800
CR2	1.088	(0.230–5.140)		1.058	(0.688–1.626)	
CR3 or more	2.042	(0.651–6.786)				
Never treated	0.329	(0.070–1.555)				

*adj* adjusted; *ALL* acute lymphoblastic leukemia; *AML* acute myeloid leukemia; *AUC_day1* area under the curve for BU after the first dose of BU; *95% CI* 95% confidence interval; CRisk, competing risk analysis of the cumulative incidence of relapse with competing event death; *CRRisk* multivariate competing risk regression analysis that is presented with Fine–Gray proportional hazard ratios (*HR*); CR1, first complete remission; CR2, second complete remission; CR3, third complete remission; HR, hazard ratio; disease status; *CumAUC*, cumulative area under the curve for BU; *MDS* myelodysplastic syndrome

“*CR3 or more*” included all patients either in CR3 or more or in partial remission or those with > 10% of circulating myeloblasts before conditioning

*Intensity of conditioning, two alkylating agents (busulfan with cyclophosphamide or melphalan) and three agents (busulfan/cyclophosphamide with melphalan or etoposide)

^#^*CumAUC* was calculated after 16 doses administered in 6 h intervals and is presented in mg*h/L of which one dose 3.7 mg*h/L is equivalent to 900 μM × min and 6.16 mg*h/L is equivalent to 1500 μM × min

*Bold*: significant *p* values below 0.05

*GSTM1-null* and *GSTT1-null* are presented as *GSTM1(−/−)* and *GSTT1(−/−)*, respectively. *GSTM1 non-null* and *GSTT1 non-null* genotypes are presented as *GSTM1(* +*)* and *GSTT1(* +*)*, respectively

Relapse was compared between *GSTM1-null* and *GSTT1-null* variants combined with other possible risk factors (Table 2B). *GSTM1/GSTT1 double null* status was independently associated with relapse with an HR of 6.52 [95% Cl, 2.76 – 15.42; *p* = 1.9 × 10^–5^]. 25%, 9.7% and 23.3% of patients with ALL, MDS and AML, respectively, were relapsed. Among them, all relapsed patients with the *GSTT1-null* genotype had ALL (Supplementary Table 3).

### LCL sensitivity to BU is associated with *GSTM1,* but not with *GSTT1* genotypes

**Fig. 2:**
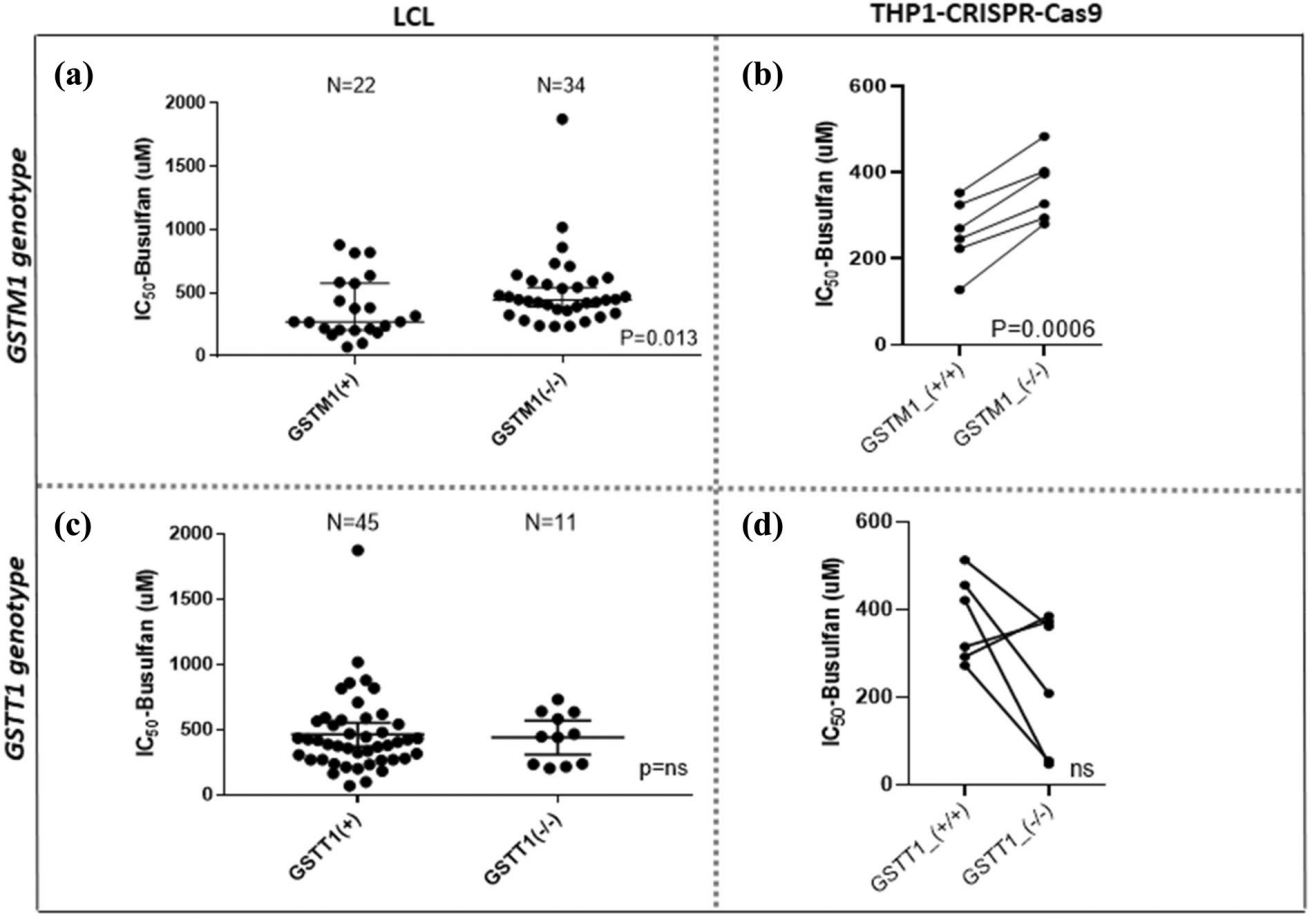
IC_50_-values for BU in *GSTM1-null* and *GSTT1-null* LCLs, THP1^*GSTM1(−/−)*^ and THP1^*GSTT1(−/−)*^ cells. IC50 values for BU were stratified according to *GSTM1-* (**A**) and *GSTT1-null* variants (**C**) in LCLs and CRISPR–Cas9 gene-edited THP1^*GSTM1(−/−)*^ (**B**) and THP1^*GSTT1(−/−)*^ (**D**) cell models. Concentration–response titration points were fitted to a Hill equation for BU. The 50% inhibitory concentrations of BU (BU-IC_50_) were determined by dose–response curve fitting using Prism 5.02 software (GraphPad SoftwareInc., CA. USA). The coefficient of determination (*R*^2^) of each plate was used to assess experimental reproducibility and was set to be above 0.95. Independent experiments were repeated at least three times. Non-parametric unpaired *t* test was used in LCLs (A, C). Pairwise comparisons by *t* test between *GST(−/−)* variants in THP1-CRISPR–Cas9 models (B., D.) were used. In THP1-CRISPR–Cas9 cell models (B., D.), dots represented are specific clones with identified *GST(−/−)* variants (+ / + vs. *−*/*−*) based on Sanger DNA-sequencing. *p* values below 0.05 were considered statistically significant. ns, not significant. *GSTM1-null* and *GSTT1-null* are presented as *GSTM1(−/−)* and *GSTT1(−/−)*, respectively. *GSTM1 non-null* and *GSTT1 non-null* genotypes are presented as *GSTM1(* +*)* and *GSTT1(* +*)*, respectively

**Fig. 3:**
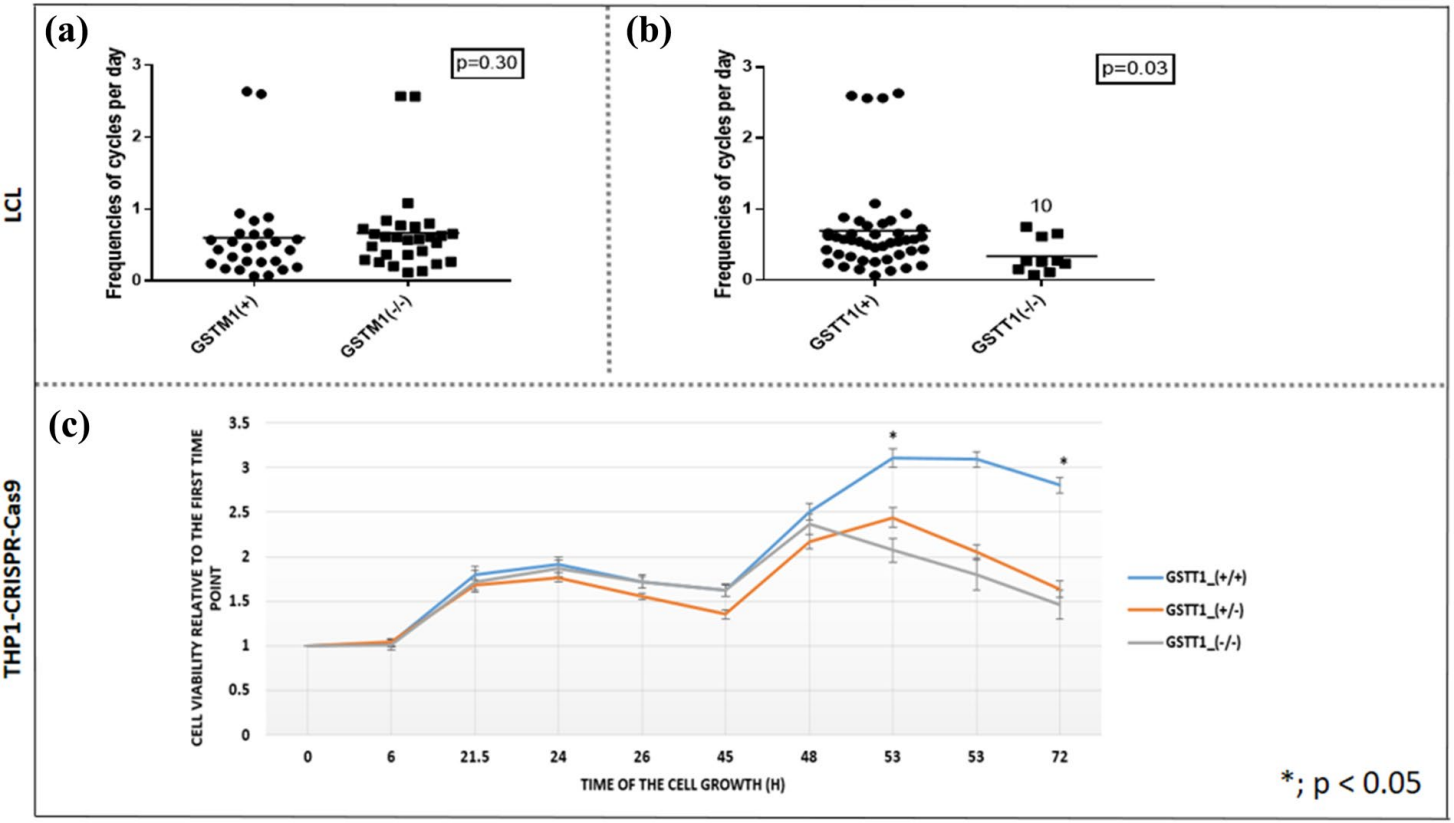
Baseline cell growth against *GSTM1-null* and *GSTT1-null* genotypes in LCLs and THP1^*GSTT1(−/−)*^ cells. Baseline cell growth was assessed against *GSTM1-null* (**A**) and *GSTT1-null* (**B**) genotypes in LCLs at 48 h end-point analysis and the (**C**) RealTime Cell Viability assay–Kinetics plot was performed for 72 h stratified by *GSTT1-null* genotype at baseline (proliferation profile) in THP1^*GSTT1(−/−)*^ cell models. **(A**, **B**) On the y-axis, the basal cell growth rate (*r*) was calculated using the following formula, appropriate for the usual exponential kinetics of cell growth (N; the number of cells) after the defined time (t; h): N_t_ = N_o_.2^tr^. (**C**) Measurement of baseline reducing the potential of viable cells according to *GSTT1(−/−)* variant in CRISPR–Cas9 gene-edited cell models was performed. The unpaired *t* test between GST genotypes in LCLs (A., B.) and Pairwise comparisons by *t* test between *GSTT1* genotypes in THP1-CRISPR–Cas9 models were used. *p* values below 0.05 were considered statistically significant. *GSTM1-null* and *GSTT1-null* are presented as *GSTM1(−/−)* and *GSTT1(−/−)*, respectively. *GSTM1 non-null* and *GSTT1 non-null* genotypes are presented as *GSTM1(* +*)* and *GSTT1(* +*)*, respectively

Significantly higher cell viability after treatment with BU was observed in LCLs with *GSTM1-null* genotype (1.8-fold, *p* = 0.013) and THP1^*GSTM1(−/−)*^ cells (1.5-fold, *p* = 0.0006) compared to *GSTM1-non-null* by 48 h end point (Fig. 2A, B, respectively) and the results were confirmed by 72-h kinetic measurements in LCLs (Supplementary Fig. 3). *GSTT1-null*, alone or in combination with *GSTM1-null,* did not show a significant association with BU-IC_50_ in LCLs and THP1^*GSTT1(−/−)*^ cell lines (Fig. 2C, D, respectively). No difference in baseline cell proliferation was seen between *GSTM1-null* and *GSTM1- non-null* cells (Fig. 3A), while the proliferation of *GSTT1-null* cells was significantly decreased in comparison to *GSTT1-non-null* carriers in LCLs carrying *GSTT1-null* genotype and THP1^*GSTT1(−/−)*^ [*p* = 0.03 (LCL, 48 h end-point measurement, Fig. 3B) and *p* < 0.05 (THP1^*GSTT1(−/−)*^, 72 h kinetic plot, Fig. 3C)].

### *GSTM1-null* genotype is associated with increased apoptosis and decreased primary necrosis after BU treatment

**Fig. 4:**
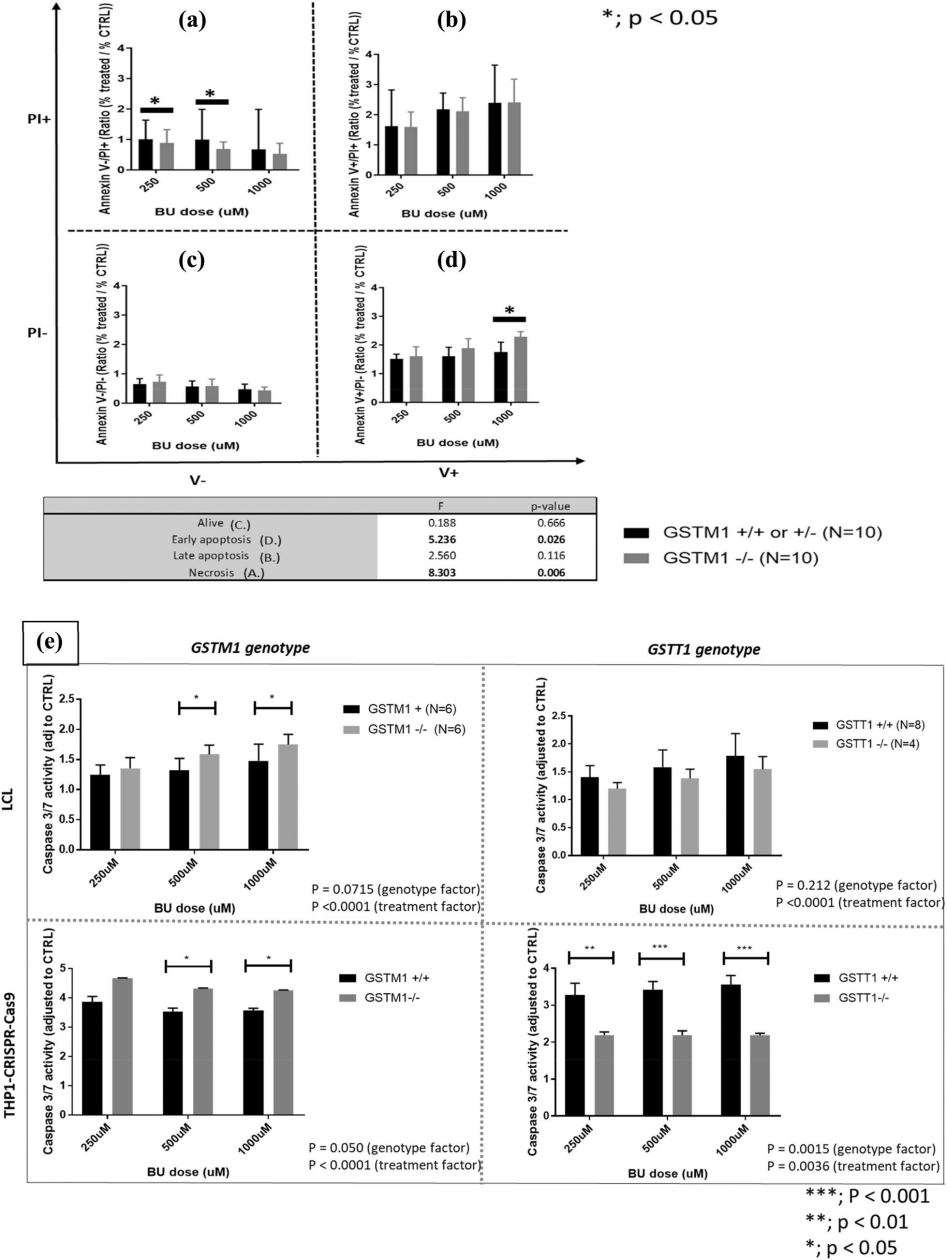
Number of necrotic and apoptotic cells in LCLs and caspase 3/7 activity in LCLs, THP1^*GSTM1(−/−)*^ and THP1^*GSTT1(−/−)*^ cells. Flow cytometric analysis (FACS) by using Annexin V/PI assay was used to assess primary necrosis (**A**), late apoptosis (**B**), live cells (**C**) and early apoptosis (**D**) in LCLs stratified according to *GSTM1-null* variants; and caspase 3/7 activity (**E**) in LCLs and THP-CRISPR–Cas9 models stratified according to *GST-null* variants at 250, 500 and 1000 μM BU 48 h post-treatment. Statistical analysis was performed by two-way ANOVA considering 250, 500 and 1000 μM BU concentrations (genotype and treatment factors); *t* tests between *GST(−/−)* variants in each condition separately were used; no statistically significant differences were observed between *GSTM1* + and *GSTM1(−/−)* LCLs and THP-CRISPR–Cas9 models in either 1% DMSO or medium only; *p* values below 0.05 were considered statistically significant. *GSTM1-null* and *GSTT1-null* are presented as *GSTM1(−/−)* and *GSTT1(−/−)*, respectively. *GSTM1 non-null* and *GSTT1 non-null* genotypes are presented as *GSTM1(* +*)* and *GSTT1(* +*)*, respectively

In a subgroup of ten *GSTM1-null* LCLs, we observed increased early apoptosis and a decreased primary necrosis (*p* = 0.026 and 0.006, respectively) at 48 h post-treatment with 250, 500 and 1000 µM of BU in comparison to ten *GSTM1-non-null* LCLs (Fig. 4A–D). No significant differences between both *GSTM1* genotype groups were observed for the number of live cells and apoptotic cells at a later stage (the mix of necrotic and real apoptotic cells). Apoptosis was further assessed through measurement of caspase 3/7 activity according to *GST(−/−)* variants showing significantly higher activation in *GSTM1-null* LCLs and THP1^*GSTM1(−/−)*^ at 250, 500 and 1000 µM BU in comparison to *GSTM1-non-null* cells (*p* < 0.05; Fig. 4E), while no differences were observed at baseline. BU-induced activation of caspase 3/7 was not significant in *GSTT1-null* LCLs (*p* = 0.21), while in THP1^*GSTT1(−/−)*^ was significantly decreased (*p* = 0.002; Fig. 4E) in comparison to *GSTT1-non-null* cells.

Cell death mechanisms were further followed by kinetic plots. We observed higher apoptotic rates in *GSTM1-null* cells (THP1^*GSTM1(−/−)*^, LCLs) through the whole 72 h of follow-up after BU treatment when compared to *GSTM1-non-null* cells from unrelated individuals (Supplementary Figs. 4A and 4C, *p* < 0.0001 and *p* = 2.6E-05, respectively). In contrast, we observed lower necrotic rates in *GSTM1-null* cells (THP1^*GSTM1(−/−)*^, LCLs) that were increasing after 26 h of BU treatment when compared to *GSTM1-non-null* cells (Supplementary Figs. 4B, D, *p* < 0.001 and 1.4E-05, respectively). Apoptosis at the same time points was lower in these cells accounting for the faster cell death, mainly as a result of primary necrosis.

### [GSSG/GSH_T_] ratios are higher in *GSTM1-non-null* LCLs and THP1^*GSTM1(*+*/*+*)*^ cells after BU treatment compared to *GSTM1-null* while total GSH levels remain unchanged

**Fig. 5:**
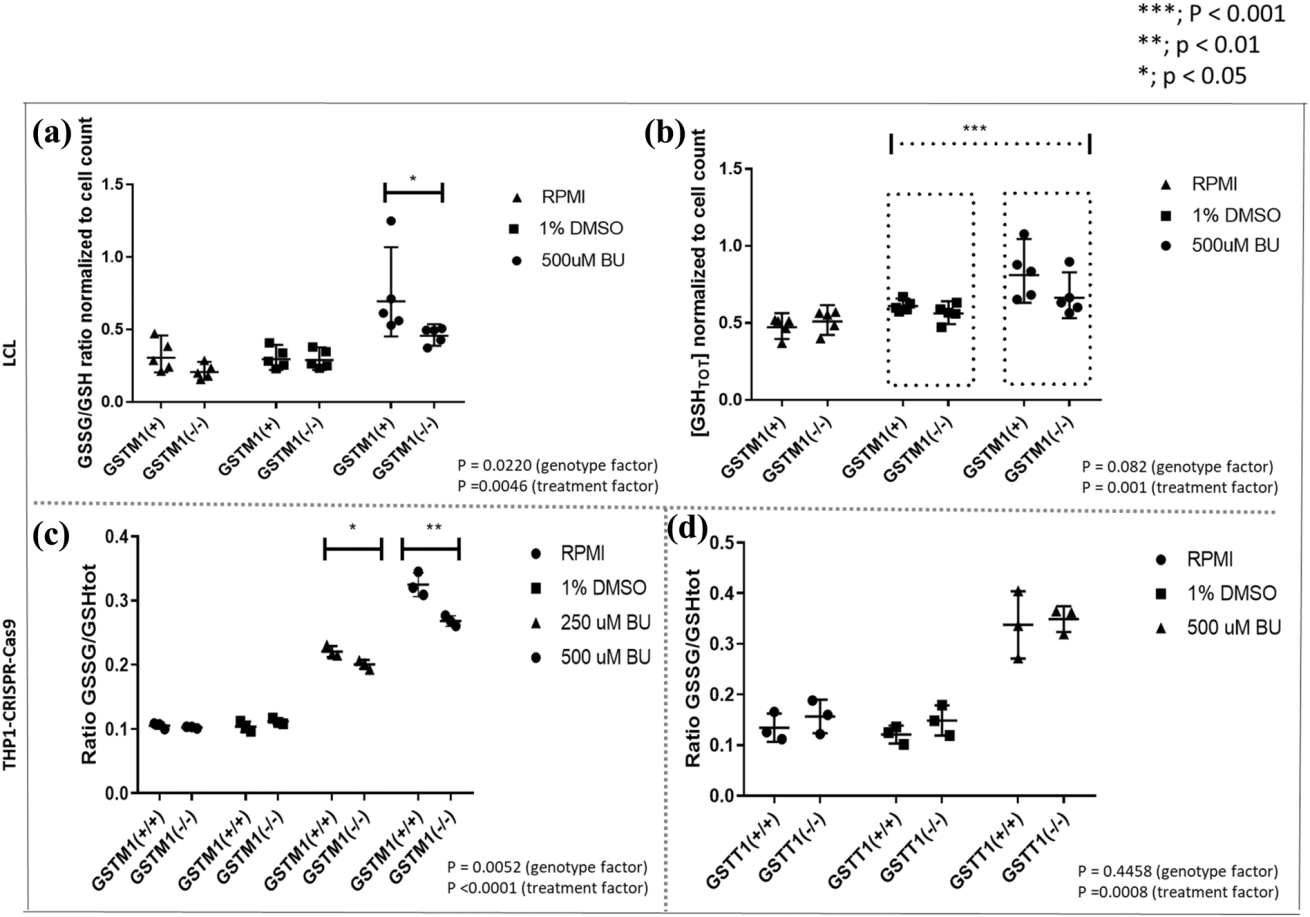
Glutathione levels in LCLs stratified according to *GSTM1-null* variant, THP1^*GSTM1(−/−)*^ and THP1^*GSTT1(−/−)*^ cells. [GSSG/GSH_T_] ratio (**A**) and GSH_T_ (**B**) in LCLs were calculated stratified according to *GSTM1-null* variant; and [GSSG/GSH_T_] ratios in CRISPR–Cas9 edited THP1^*GSTM1(−/−)*^ (**C**) and THP1^*GSTT1(−/−)*^ (**D**) cell models after the treatment with 500 μM BU. Statistical analysis was performed by the two-way ANOVA considering 250 or 500 μM BU concentration (genotype factor); *t* tests between *GST(−/−)* variants in each condition separately were used; no statistically significant differences were observed between *GST(−/−)* variants in LCLs and THP-CRISPR–Cas9 models in either 1% DMSO or medium; *p* values below 0.05 were considered statistically significant. *GSTM1-null* and *GSTT1-null* are presented as *GSTM1(−/−)* and *GSTT1(−/−)*, respectively. *GSTM1 non-null* and *GSTT1 non-null* genotypes are presented as *GSTM1(* +*)* and *GSTT1(* +*)*, respectively

At baseline, no differences in [GSSG/GSH_T_] ratios were observed between *null* and *non-null* LCLs and THP1 cells for the *GSTM1* gene. However, 48 h after 500 μM BU treatment, [GSSG/GSH_T_] ratios were increased 1.6- (*p* = 0.02, LCLs) and 1.3-fold (*p* = 0.005, THP1) in *GSTM1 non-null* compared to *GSTM1-null* (Fig. 5A, C, respectively). A similar trend was observed after the 250 μM BU treatment (48 h) in THP1^*GSTM1(−/−)*^ in comparison to THP1^*GSTM1(*+*/*+*)*^ cells (Fig. 5C). In THP1, no significant difference was observed according to *GSTT1* genotype after the treatment with BU or at baseline (Fig. 5D). In addition, we observed a significant increase in total GSH levels after the 500 μM BU treatment, irrespective of the *GSTM1* genotype (Fig. 5B, *p*=0.001), thus indicating the potential for BU-related induction of GSH synthesis.

## Discussion

Our clinical association study performed in 86 children with HMs undergoing HSCT following BU-based conditioning regimens demonstrated that patients harboring homozygous deletions in both *GSTM1* and *GSTT1* genes presented a high risk of relapse (HR_relapse_ 7.2 [95% Cl, 2.2–23.9; *p* = 0.002]) and a lower EFS (HR_EFS_ 4.092 [95% Cl, 1.829–9.152; *p* = 0.001]). After adjustment for known risk factors (diagnosis, disease status, the intensity of conditioning regimen and BU exposure), the association remained significant demonstrating that the deletion of both *GST* genes is an independent risk factor for relapse (adjusted HR 6.52 [95% CI, 2.8–15.4; *p* = 1.9 × 10^–5^]). Although it is a small cohort, this is the first report on the risk of post-HSCT relapse in relation to the germline *GSTM1-* and *GSTT1-null* variants in children with HMs. Until now, only one study conducted in BU/CY-based HSCT settings although in adults showed increased relapse rates in patients carrying *GSTM1-null* genotype, while no association was identified with *GSTT1-null* genotype (Terakura et al. 2020). Concerning non-transplant-based studies in pediatric or adult patients, a similar association between *GSTM1/GSTT1 double null* carriers and increased risk of relapse (Barragan et al. 2007; Borst et al. 2012; Takanashi et al. 2003, Xiao Q. et al. 2014), lower complete remission rate (Xiao Z. et al. 2008) and lower EFS were demonstrated (Chen et al. 1997; Hall et al. 1994; Leonardi et al. 2017; Rocha et al. 2005; Stanulla et al. 2000; Takanashi et al. 2003; Teachey and Hunger 2013; Woo et al. 2000; Zhang et al. 2017). There are nevertheless a few studies showing no such association (Franca et al. 2012; Zareifar et al. 2013), in which the small number of patients or the different treatment regimens may have mainly precluded defining a relationship between GST variants.

Based on the known detoxifying role of GSTs, our results from the clinical association are contradictory. Although GSTA1 is the main enzyme involved in BU detoxification, GSTM1 is also highly expressed in the liver and recognized as involved in BU conjugation (Ansari et al. 2017; Bremer et al. 2015; Czerwinski et al. 1996; Kim et al. 2011), precluding the BU to cross-link with the DNA strands. Functional variants of the genes coding for GSTs may then interfere in HSCT by affecting BU metabolism. It is known that low BU exposure (CumAUC < 59 mg × h/L) is associated with graft failure and relapse (Ansari et al. 2017; Bartelink et al. 2016; Philippe et al. 2016), whereas high BU exposure (CumAUC > 98.6 mg × h/L) could reduce post-HSCT relapse in leukemia at the cost of an increase in organ toxicities, and therefore transplantation-related mortality (Ansari et al. 2017; Bartelink et al. 2016; McCune et al. 2002; McCune and Holmberg 2009; Philippe et al. 2016). However, at the level of HCs, less is known about the direct effect of BU.

We compared BU-related cell death mechanisms in LCLs and THP1 with and without *GSTM1* and/or *GSTT1* genes after exposure to BU. LCLs were chosen as the in vitro model to resemble heterogeneity seen in a clinical cohort. Since each LCL is derived from a different individual genetic background, the studied effect due to BU is specifically related to the *GSTM1*- and *GSTT1-null* and *–non-null* genotypes. The acute monocytic leukemia (THP1) cell model was selected for the production of the CRISPR–Cas9 GSTM1- and/or GSTT1 knockout model, as we have the most patients with a relapsed AML (Supplementary Table 3). Selection of these two cell models aids in evaluating BU-dependent and -independent *GSTT1* and *GSTM1* effects. We demonstrated that only *GSTM1-null* (but not *GSTT1-null*) is associated with higher resistance to BU as determined by higher BU-IC_50_ values of *GSTM1-null* LCLs and THP1^*(GSTM1−/−)*^ in comparison to *GSTM1-non-null* cells. This could be due to a change in the redox equilibrium as demonstrated by lower levels of oxidized GSH, lower primary necrosis and higher early apoptosis. An increase of *GSTM1-null* LCL’s viability was confirmed either by continuous follow-up of redox potential within 72 h. Apoptosis/necrosis kinetic results demonstrate that BU-induced apoptotic processes are more pronounced in *GSTM1-null* LCLs. In contrast, primary necrotic cell death was more pronounced in *GSTM1-non-null* cells when comparing with the *GSTM1-null* cells. In addition, primary necrosis was significantly induced at an earlier stage in *GSTM1-non-null* cells. These results show that *GSTM1-null* variants can modulate BU-induced cell death, which were supplemented further by increased activation of known apoptotic markers caspase-3 or -7 in *GSTM1-null* LCLs and THP1 in comparison to *GSTM1-non-null* cells. Importantly, observed reduced rates of *GSTM1*-dependent cell death cannot be attributed to the increased baseline cell proliferation.

The findings of higher primary necrosis, lower early apoptosis and lower cell viability in *GSTM1-non-null* HCs compared to *GSTM1-null* cells treated with BU were unexpected. Contrary to our observations, many studies showed associations between increased expression or activity of GSTs and resistance mechanisms against a range of cytotoxic drugs (Hoban et al. 1992; Smith et al. 1989). These results could potentially be explained by not only direct detoxification with GSH, but also through negative regulation of pro-apoptotic protein kinases, such as apoptosis signal-regulating kinase 1 (ASK1) (Board and Menon 2013; Tew and Townsend 2012). For instance, stress conditions cause the release of ASK1 from GSTM1, thereby leading to induction of apoptosis, which was shown in our experiments after induction with BU. In addition, *GSTM1-null* cells carrying more free ASK1 for phosphorylation activation are expected to have more apoptosis upon BU-induced stress in comparison to *GSTM1-null* cells which is in accordance with our in vitro results.

However, the observed paradox in increased cell death of GSTM1 well-expressed cells upon BU treatment could additionally be explained by findings of the study of DeLeve et al. (2000), demonstrating that in murine hepatocytes BU is cytotoxic also through oxidative stress caused by BU metabolites (BU glutathione S-conjugate thiophenium ion, GS^+^THT) and by the depletion of GSH in addition to DNA alkylation. The toxic metabolites of BU/GSH metabolism are mainly oxidized by flavin-containing monooxygenases (FMOs, e.g., FMO3) and cytochromes (CYPs, e.g., CYP3A4) (El-Serafi et al. 2017) to water-soluble non-toxic metabolites [e.g., sulfolane (Uppugunduri et al. 2017)]. However, CYP3A4 and FMO3 are mainly expressed in the liver (accounting for 54% of overall tetrahydrothiophene [THT] disappearance, the metabolite of BU), and less in LCLs, as observed in our laboratory (data not shown) and by others (https://www.proteinatlas.org). After RNA sequencing in LCLs, very low or no gene expressions of *CYP 2D6, 2C19, 2C9, 2B6, 2C8, 4A11, 3A4, FMO1* and *FMO3* were identified. In this context, the oxidative burst caused by electrophilic molecules from BU–GSH conjugation (Udensi and Tchounwou 2014; Zmorzynski et al. 2015) in addition to the absence of *CYP3A4* and *FMO3* could be a reason for the lower sensitivity of *GSTM1-null* HCs to BU, as observed in LCLs and THP1. In contrast, higher total expressions of *CYPs* and *FMOs* in hepatocytes (El-Serafi et al. 2017) could explain why *GSTA1*-slow BU metabolizing individuals in addition to the absence of GSTM1 activity show potentially more treatment-related toxicities [e.g., SOS (Srivastava et al. 2004) and aGvHD (Elhasid et al. 2010)] than carriers with normal GST’s enzyme activities. A hypothetical comparative model of the difference in BU fate between hepatocytes and lymphocytes is suggested in Supplementary Fig. 5 and warrants further investigation.

The genetically determined different cell fate after BU exposure might explain the apparently discordant results between the relapse incidence in patients carrying *GSTM1-null* genotype (in combination with *GSTT1-null*) and the cellular resistance to BU in *GSTM1-null* LCLs and THP1^*GSTM1(−/−)*^. The higher rates of necrosis in *GSTM1*-*non-null* cells might predict a pro-inflammatory cell death of the malignant cells, resulting in enhanced immunogenicity (Sachet et al. 2017). Unlike the other chemotherapeutic regimens including autologous transplantation, the efficacy of the allogeneic transplantation relies on the graft-versus-leukemia effect, especially in HMs (Horowitz et al. 1990; Yeshurun et al. 2019), but that theory should be further explored.

Another relevant observation is the significantly increased post-HSCT relapse in *GSTT1-null* when combined with *GSTM1-null* genotype in children with HMs. The link between GSTT1 and post-HSCT relapse is not clear yet. Our in vitro observations cannot be attributed to the BU-related differences in IC_50_ values or [GSSG/GSH_T_] ratios. Other pharmacogenomics studies also demonstrated that genetic variations in *GSTT1* are not associated with BU clearance or liver toxicity (Gaziev et al. 2010; Goekkurt et al. 2007; Kim et al. 2011; Srivastava et al. 2004). Nevertheless, we observed faster baseline proliferation in *GSTT1-non-null* LCLs/THP1 and a slightly higher baseline increase of caspase 3/7 activation compared to those with *GSTT1-null* genotype, indicating GSTT1 potential involvement of BU- independent mechanisms in the relapse development.

The results of the present clinical study are limited by the retrospective study design and relatively small pediatric sample size with no clinical validation cohort. However, the sample size of 86 patients has at least 80% power with 10% of observed combined *GSTM1/GSTT1 double null* variants’ frequency and relapse incidence with the estimated observed effect size of ≥ 7.0 and alpha value of 0.05. The primary diagnosis of HMs was made at the referring institution and was not centrally reviewed. Well-known risk factors such as somatic genetic/cytogenetics abnormalities, the donor DNA and the initial response to the treatment (e.g., MRD) were not available. However, as described in Supplementary Table 2, similar characteristics were present between the *GST* genetic subgroups (*p* values > 0.05). The *GST-null* variants were not associated with the status of the disease before HSCT and we assume that the germline genotype impact on protein expression was present in malignant cells as shown by Weiss et al. (2007). The majority of cases in our study underwent a BU–CY conditioning regimen; however, it is not known if this association is specific to a BU–CY conditioning regimen only or unspecific to other chemotherapeutics used in the HSCT setting (e.g., Thio or Mel) (Hao et al. 2020). For instance, active metabolites of CY (e.g., acrolein) are also eliminated by GSH conjugation catalyzed by GSTs (Uppugunduri et al. 2017). This needs to be evaluated in the future with a focus on whether GSTs play a major role in determining clinical outcomes. This aspect is currently being evaluated by our group using a cohort from multiple centers with the usage of multiple conditioning regimens. Furthermore, the transplant-related mortality or combined toxicities were not associated with the *GSTM1-* and *GSTT1-null* variants (data are not shown), suggesting compensation of BU conjugation by other GSTs, especially GSTA1, which is mainly expressed in hepatocytes and other somatic cells.

## Conclusions

In summary, we report that *GSTM1/GSTT1 double null* genotypes could serve as genetic biomarkers for identifying pediatric patients with HMs at higher risk of relapse after an allogeneic HSCT following BU-containing conditioning. On the other hand, the absence of those markers might predict the patients who more likely will respond to the chemotherapy-based conditioning. Functional studies indicated different mechanisms of cell death upon exposure to BU based on the presence or absence of *GST-null* alleles and the in vivo impact of those findings must be further explored.

## Supplementary Information

Below is the link to the electronic supplementary material.

Supplementary file1 (DOCX 1188 KB)

## Data Availability

All data presented are provided freely in this manuscript including any supplementary data. The raw datasets used and/or analyzed during the current study are available from the corresponding author on reasonable request.
