# Correction to: *GSTM1* and *GSTT1 double null* genotypes determining cell fate and proliferation as potential risk factors of relapse in children with hematological malignancies after hematopoietic stem cell transplantation

**DOI:** 10.1007/s00432-021-03830-0

**Published:** 2021-10-30

**Authors:** Simona Jurkovic Mlakar, Satyanarayana Chakradhara Rao Uppugunduri, Tiago Nava, Vid Mlakar, Hadrien Golay, Shannon Robin, Nicolas Waespe, Mohamed Aziz Rezgui, Yves Chalandon, Jaap Jan Boelens, Robert G. M. Bredius, Jean-Hugues Dalle, Christina Peters, Selim Corbacioglu, Henrique Bittencourt, Maja Krajinovic, Marc Ansari, the paediatric diseases working party of the European society for blood and marrow transplantation

**Affiliations:** 1 CANSEARCH Research Platform in Paediatric Oncology and Haematology, Department of Paediatrics, Gynecology and Obstetrics, Faculty of Medicine, University of Geneva, Geneva, Switzerland; 2 Division of Paediatric Oncology and Haematology, Department of Women, Children and Adolescents, Pediatric Oncology and Hematology Unit, Geneva University Hospitals and University of Geneva, Rue Willy-Donzé 6; Bureau 5-507, 1211 Genève, Switzerland; 3 Institute of Social and Preventive Medicine, University of Bern, Bern, Switzerland; 4 Charles-Bruneau Cancer Center, CHU Sainte-Justine Research Center, Montreal, QC Canada; 5 Division of Haematology, Department of Oncology, Geneva University Hospital and Faculty of Medicine, University of Geneva, Geneva, Switzerland; 6 Paediatric Blood and Marrow Transplantation Program, University Medical Center Utrecht, Utrecht, The Netherlands; 7 Department of Pediatrics, Leiden University Medical Center, Leiden, The Netherlands; 8 Paediatric Haematology Department, Robert Debré Hospital, Assistance Publique, Hôpitaux de Paris, Paris, France; 9 St Anna Children’s Hospital, Department of Pediatrics, Medical University Vienna, Vienna, Austria; 10 Department of Pediatric Hematology, Oncology and Stem Cell Transplantation, University of Regensburg, Regensburg, Germany; 11 Department of Paediatrics, Faculty of Medicine, University of Montreal, Montreal, QC Canada; 12 Present Address: Clinical Pharmacology Unit, CHU Sainte-Justine, Montreal, QC Canada; 13 Department of Pharmacology, Faculty of Medicine, University of Montreal, Montreal, QC Canada

## Correction to: Journal of Cancer Research and Clinical Oncology 10.1007/s00432-021-03769-2

In the original article published, there are several corrections in the author names published in the author group. The corrections are mentioned below:

The given name of the 1st author is Simona and the family name is Jurkovic Mlakar.

The first name of the 2nd author is Satyanarayana Chakradhara Rao and the family name is Uppugunduri.

The author name Mohammed Aziz Rezgui incorrectly published as Mohamed-Ali Rezgui.

In addition, the affiliation number 2 has been changed from University Hospital of Geneva into “Geneva University Hospitals and University of Geneva”, because of just recent official changes at the university.

The original article has been corrected.

