# Supplementary material for: GSTM1 and GSTT1 double null genotypes determining cell fate and proliferation as potential risk factors of relapse in children with hematological malignancies after hematopoietic stem cell transplantation
Source: J Cancer Res Clin Oncol. 2021 Sep 9;148(1):71–86. doi: 10.1007/s00432-021-03769-2 (PMC8752561; doi:10.1007/s00432-021-03769-2)
Supplement: Supplementary file 1 — Supplementary file1 (DOCX 1188 KB) [file 432_2021_3769_MOESM1_ESM.docx]

**Supplementary Material**

***GSTM1* and *GSTT1 double null* genotypes determining cell fate and proliferation as potential risk factors of relapse in children with hematological malignancies after hematopoietic stem cell transplantation.**

On Behalf of the Pediatric Disease Working Party of the European Society for Blood and Marrow Transplantation

**AUTHORS:**

Simona Jurkovic Mlakar ^1,2^, Chakradhara Rao Uppugunduri Satyanarayana ^1,2^, Tiago Nava ^1,2^, Vid Mlakar ^1,2^, Hadrien Golay^1,2^, Shannon Robin ^1,2^, Nicolas Waespe ^1,3^, Mohamed Aziz Rezgui ^4^, Yves Chalandon ^5^, Jaap Jan Boelens ^6^, Robert G.M. Bredius ^7^, Jean-Hugues Dalle ^8^, Christina Peters ^9^, Selim Corbacioglu ^10^, Henrique Bittencourt ^4, 11-13^, Maja Krajinovic ^4, 11-13^ and Marc Ansari ^1,2^

**Supplementary Table 1.** List of Lymphoblastoid Cell Lines as used for the particular functional cell assay

* LCL name; each code starts with GM before the number. *GSTM1-null* and *GSTT1-null* are presented as *GSTM1(-/-)* and *GSTT1(-/-)*, respectively. *GSTM1 non-null* and *GSTT1 non-null* genotypes are presented as *GSTM1(+)* and *GSTT1(+)*, respectively.

**Supplementary Table 2.** Patient and transplant characteristics at the time of HSCT according to *GSTM1-null* and *GSTT1-null* genotypes

ND, no data; ALL, acute lymphoblast leukemia; AML, acute myeloid leukemia; MDS, myelodysplastic syndrome; CR1, first complete remission; CR2, second complete remission; CR3, third complete remission; BM, bone marrow; CB, cord blood; PBSCs, peripheral blood stem cells; MMUD, non-identical unrelated; MMRD, non-identical related; MUD, identical unrelated; MRD, genotype HLA identical sibling; *disease phase "CR3 or more" included all patients either in CR3 or more or in partial remission or those with >10% of circulating myeloblasts before conditioning; #, 2 alkylating agents (busulfan with cyclophopshamide or melphalan) and 3 agents (busulfan/cyclophopshamide with melphalan or etoposide);

Frequencies of only patients carrying deletion genotype (*GSTM1(-/-)*, *GSTT1(-/-)* and *GSTM1(-/-)/GSTT(-/-)* are presented; *GSTM1-null* and *GSTT1-null* are presented as *GSTM1(-/-)* and *GSTT1(-/-)*, respectively. *GSTM1 non-null* and *GSTT1 non-null* genotypes are presented as *GSTM1(+)* and *GSTT1(+)*, respectively.

**p-value* was obtained by Pearson Chi-Square test; p-value below than 0.05 was considered statistically significant.

**Supplementary Table 3.** Type of tumor of relapsed patients according to *GSTs* genotype subgroups

% in brackets (1^st^ column) is calculated as: N of patients with relapsed tumor divided by total N of patients with malignancies; 2^nd^, 3^rd^, 4^th^, 5^th^, 6^th^ columns, N of relapsed patients with specific genotype divided by N of total relapsed patients with a specific type of tumor

ALL, acute lymphoblast leukemia; MDS, myelodysplastic syndrome; AML, acute myeloid leukemia; N, number of patients

Frequencies of only patients carrying deletion genotype (*GSTM1(-/-)*, *GSTT1(-/-)* and *GSTM1(-/-)/GSTT(-/-)* are presented; *GSTM1-null* and *GSTT1-null* are presented as *GSTM1(-/-)* and *GSTT1(-/-)*, respectively. *GSTM1 non-null* and *GSTT1 non-null* genotypes are presented as *GSTM1(+)* and *GSTT1(+)*, respectively.


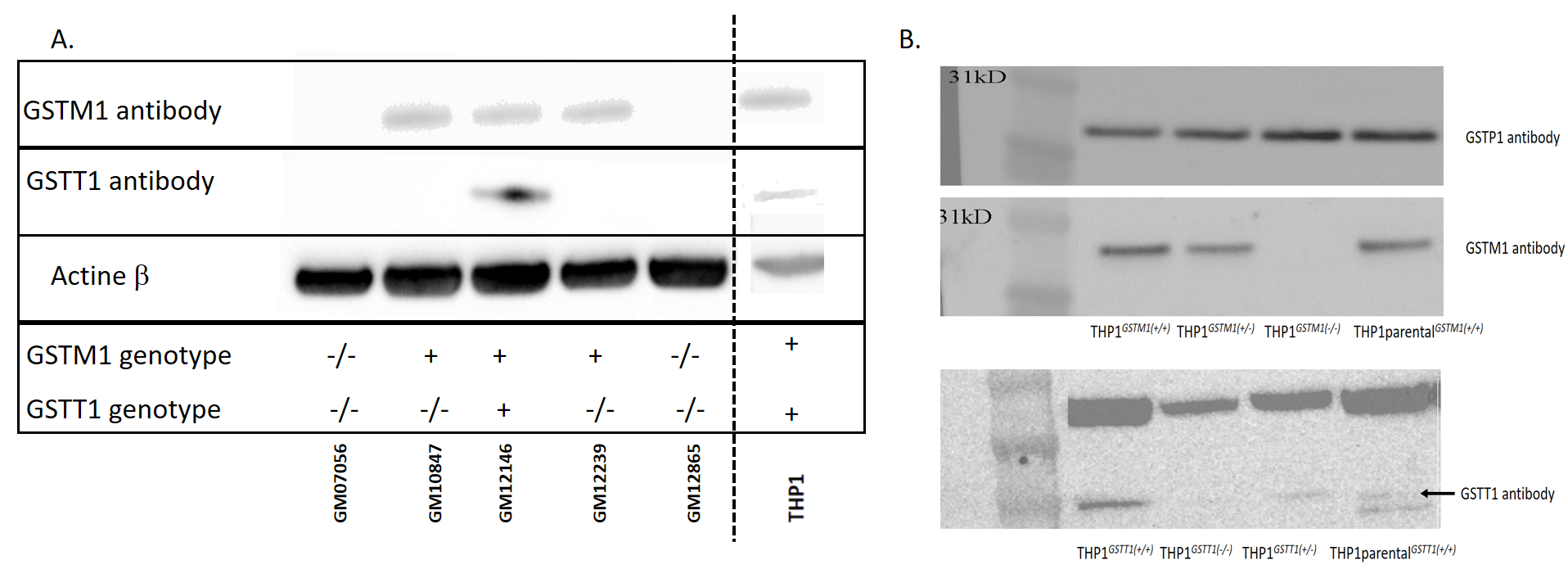
**Supplementary figure 1. Cell models characterization** A. Western blot analysis for the presence of GSTM1 and GSTT1 proteins in LCLs and THP1; B. GSTM1 and GSTT1 protein expression and C. GST activity in CRISPR-Cas9 gene-edited THP1 cell models

C.

Due to the consistency of results, GSTM1 and GSTT1 Western blots of the remained LCLs are not presented; Actine ß (**A**) and GSTP1 proteins (**B**) are used as controls. (**C**) Paired t-test was performed for the comparison of GST activities between following cell models: THP1*^GSTM1(-/-)^* vs THP1*^GSTM1(+/+)^*; THP1*^GSTT1(-/-)^* vs THP1*^GSTT1(+/+)^*; each based on five independent experiments. A p-value below 0.05 was considered statistically significant.

**Supplementary figure 2.** Relationship between GST(-/-) variants with cumulative incidences of event-free survival (EFS) (A) and overall survival (OS) (B) in univariate Cox-regression analyses (data are presented by 1-Kaplan Meier curves)


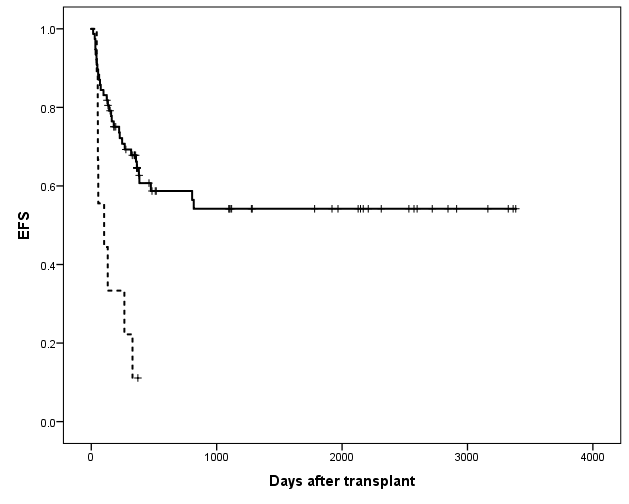


**4.092 (95%Cl, 1.829-9.152; p<0.001)**

A.

***GSTM1(+)/GSTT1(+); 31/77***

***GSTM1(-/-)/GSTT1(-/-); 8/9***

B.


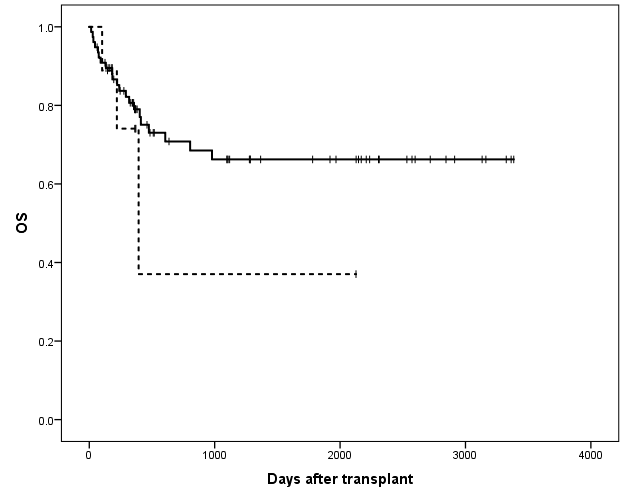


***GSTM1(-/-)/GSTT1(-/-);3/9***

***GSTM1(+)/GSTT1(+);21/77***

**1.689 (95%Cl, 0.497-5.743; p=0.401)**

**Supplementary figure 3.** RealTime Cell Viability assay- Kinetics plot BU72h of LCLs stratified according to *GSTM1-null* genotype at BU concentrations of 100, 250 and 500 μM

**
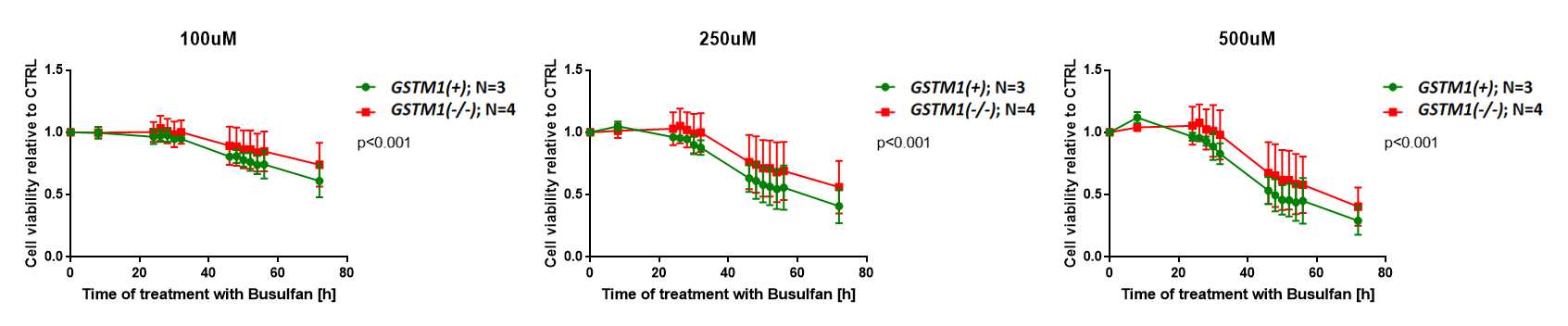
**

Independent experiments were repeated two- times. Error bars represent the standard deviations. Wilcoxon matched-pairs signed-rank test between *GSTM1 genotypes* in LCLs was used. P-values below 0.05 were considered statistically significant; N, number of cell lines;

*GSTM1-null* and *GSTT1-null* are presented as *GSTM1(-/-)* and *GSTT1(-/-)*, respectively. *GSTM1 non-null* and *GSTT1 non-null* genotypes are presented as *GSTM1(+)* and *GSTT1(+)*, respectively.

**Supplementary figure 4.** Real-time Apoptosis ([A] THP1, [C] LCLs) –Necrosis ([B] THP1, [D] LCLs) kinetic plots after the treatment with BU stratified according to *GSTM1-null* genotype

A.

**

**

***

**

*

***

**P_apoptosis_ < 0.0001**

**

*

*

*

*

*

**

*

*

*

*

B.

*

*

*

*

*

*

*

*

**P_necrosis_ < 0.0011**

*

*


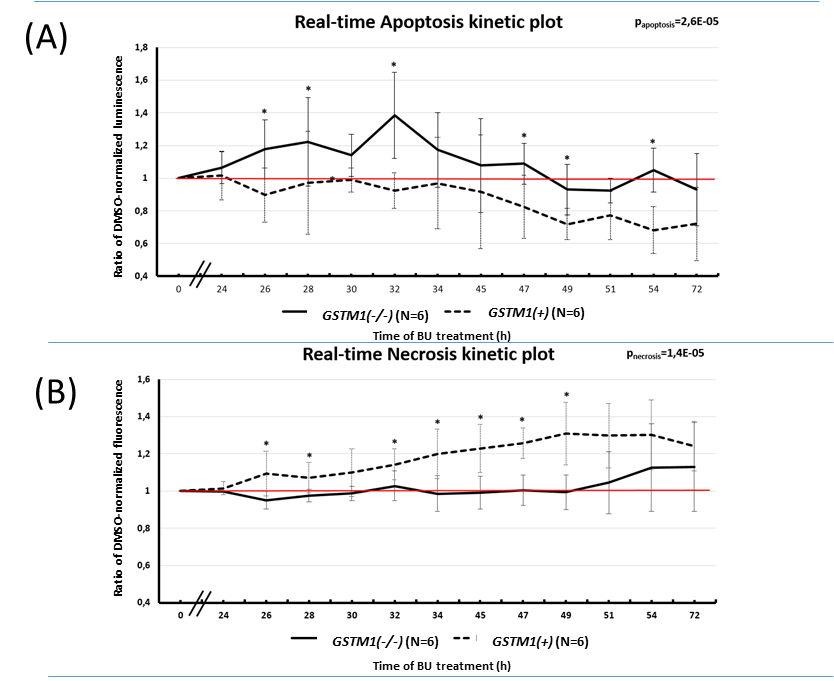


C.

D.

Independent experiments were repeated two- times. Error bars represent the standard deviations. Wilcoxon matched-pairs signed-rank test between *GSTM1 genotypes* in LCLs were used. P-values below 0.05 were considered statistically significant; t-test at each time point stratified to GSTM1 genotypes was used and was defined as the following: *<0.05; **<0.005; ***<0.0005; N, number of cell lines; Concentrations of BU for the treatment of LCLs, THP1*^GSTM1(-/-)^* and THP1*^GSTM1(+/+)^* were chosen according to their average IC_50_ as the following: 500, 350 and 250 μM, respectively.

*GSTM1-null* and *GSTT1-null* are presented as *GSTM1(-/-)* and *GSTT1(-/-)*, respectively. *GSTM1 non-null* and *GSTT1 non-null* genotypes are presented as *GSTM1(+)* and *GSTT1(+)*, respectively.


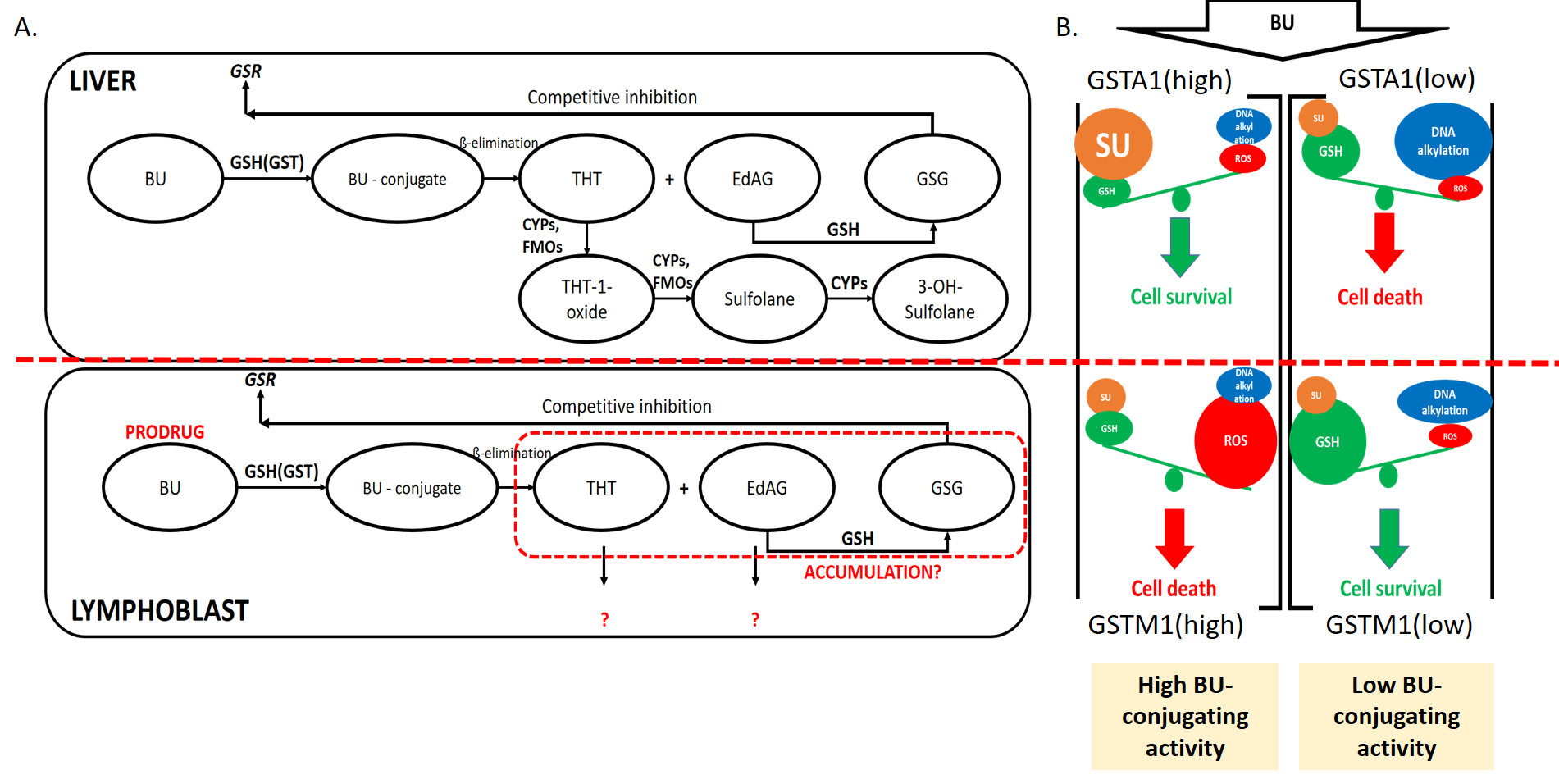
**Supplementary figure 5.** Comparison of Busulfan metabolic fates predicted in lymphoblast and hepatic cells (A) and proposed mechanisms of BU toxicity according to *GSTM1-null* genotype (B)

The mechanisms of BU toxicity are summarized based on our observations and other studies.

(**A.**) In hepatocytes, BU (1,4-butanediol dimethanesulfonate) undergoes conjugation with glutathione (GSH) catalyzed by GST (GSTA1 (glutathione-S-transferase enzyme Alpha1), GSTM1 (glutathione- S-transferase enzyme Mu 1), GSTP1 (glutathione-S-transferase enzyme Pi 1) isoenzymes). The BU-conjugate (busulfan sulfonium ion conjugate) forms THT (tetrahydrothiophene), which undergoes the following oxidation reactions to form finally sulfolane (2,3,4,5-tetrahydrothiophene-1,1-dioxide) via THT1-oxide, catalyzed by Cytochrome P 450 enzymes (CYPs), and flavin mono oxidase (FMO). Sulfolane eventually undergoes oxidation to form 3-hydroxyl sulfolane. In lymphoblasts, oxidation reactions of THT with low expressed CYPs and FMOs are decreased, potentially leading to accumulation of THT and EdAG (γ-glutamyldehydroalanylglycine) forms or activation of alternative pathways, such as mercaptopurinic acid pathway forming N-acetyl L-cysteine conjugate of sulfonium ion. EdAG condenses with GSH in a Michael addition reaction to form a lanthionine-containing thioether (GSG), which is a non-reducible analogue of glutathione disulfide (GSSG). The GSG acts as a competitive inhibitor of glutathione reductase, the enzyme necessary to convert GSSG disulfide back to GSH. Depletion of its activity can lead to the increased production of reactive oxygen species (ROS) thereby contributing further to cell damage.

(**B.**) Different proposed models of BU toxicity in hepatocytes and lymphoblasts with *GSTM1 non-null* (“high”) or *GSTM1-null* (“low”) genotypes are suggested. In hepatocytes with *high BU-conjugating activity* (e.g. GSTM1 non-null, GSTA1), a GSH activated pathway prevents the BU hepatotoxicity (better “cell survival”) as the result of further fast detoxification process of THT form with CYPs and FMOs to non-toxic sulfolane (SU) form. In the GSH-depletion pathway (or with *low BU-conjugating activity* (e.g. *GSTM1-null* cells)), more unconjugated BU is responsible for DNA alkylation, leading to increased hepatotoxicity (“cell death”). The low availability of oxidation enzymes (CYPs, FMOs) in lymphoblasts reverses the function of BU. Cell death is here more caused by oxidative stress as generated by accumulated forms THT and EdAG (or GSG) than DNA damage itself in GSTM1 non-null lymphoblasts. The role of BU here is as the prodrug and the first conjugation with GSH (e.g. in GSTM1 non-null) represents activation of its activity. *GSTM1-null* cells are more protected (better “cell survival”) due to higher availability of GSH pool and hence less activation of oxidative stress, while DNA alkylation caused by BU directly is here less important for the overall efficacy of this compound in these cells.
